# Dual-resonant scanning multiphoton microscope with ultrasound lens and resonant mirror for rapid volumetric imaging

**DOI:** 10.1038/s41598-022-27370-w

**Published:** 2023-01-04

**Authors:** Chia-Wei Hsu, Chun-Yu Lin, Yvonne Yuling Hu, Shean-Jen Chen

**Affiliations:** 1 College of Photonics, National Yang Ming Chiao Tung University, Tainan, 71150 Taiwan; 2 Department of Photonics, National Cheng Kung University, Tainan, 70101 Taiwan; 3 Taiwan Instrument Research Institute, National Applied Research Laboratories, Hsinchu, 300 Taiwan

**Keywords:** Biological techniques, Optics and photonics

## Abstract

A dual-resonant scanning multiphoton (DRSM) microscope incorporating a tunable acoustic gradient index of refraction lens with a resonant mirror is developed for high-speed volumetric imaging. In the proposed microscope, the pulse train signal of a femtosecond laser is used to trigger an embedded field programmable gate array to sample the multiphoton excited fluorescence signal at the rate of one pixel per laser pulse. It is shown that a frame rate of around 8000 Hz can be obtained in the *x–z* plane for an image region with a size of 256 × 80 pixels. Moreover, a volumetric imaging rate of over 30 Hz can be obtained for a large image volume of 343 × 343 × 120 μm^3^ with an image size of 256 × 256 × 80 voxels. Moreover, a volumetric imaging rate of over 30 Hz can be obtained for a large image volume of 256 × 256 × 80 voxels, which represents 343 × 343 × 120 μm^3^ in field-of-view. The rapid volumetric imaging rate eliminates the aliasing effect for observed temporal frequencies lower than 15 Hz. The practical feasibility of the DRSM microscope is demonstrated by observing the mushroom bodies of a drosophila brain and performing 3D dynamic observations of moving 10-μm fluorescent beads.

## Introduction

Multiphoton excited fluorescence (MPEF) microscopy has the advantages of deep penetration, low photobleaching, and minimum invasiveness, and is thus an extremely powerful tool for three-dimensional (3D) in vivo imaging in various biomedical applications^1^. Combining an ultrashort pulsed laser and a high numerical aperture (NA) objective, MPEF microscopes generate a high laser peak intensity at the focal point, which induces fluorescence excitation and facilitates volumetric imaging with a high signal-to-noise ratio (SNR) and low signal crosstalk when integrated with point-to-point scanning. Existing point-scanning MPEF microscopes generally use two galvanometer (GM) scanners to probe the sample of interest. However, such a design limits the imaging frame rate to just several frames per second (fps), which is too low for real-time imaging applications. Various studies have shown that the imaging speed can be improved by replacing the GM scanners with a resonant mirror (RM) or polygon mirror, thereby achieving a line scan rate of 10 to 30 kHz and an imaging speed of up to 30 fps for an image size of 512 × 512 pixels^2,3^.

Temporal focusing (TF) MPEF microscopy has attracted significant interest as a means of achieving widefield excitation and higher imaging frame rates through the parallelization of the imaging process^4^. For example, the present group achieved a TF imaging speed of more than 400 fps, which depends on the camera frame rate, and a volumetric imaging rate of up to 30 volumes per second (vps)^5^. However, due to widefield excitation effects, the imaging quality of MPEF microscopes is commonly degraded by scattering and signal crosstalk. Thus, even when using a high-sensitivity electron-magnifying CCD (EMCCD) as the detector, widefield microscopy is still limited to the detection of very weak fluorescent signals. Photomultiplier tube (PMT) offers a potential option for improving the sensitivity of point-scanning microscopes.

Resonant scanners and non-inertial modules provide a promising new approach for high-speed scanning microscopy. Some researchers have suggested that the imaging speed can also be enhanced by using acousto-optic deflectors to achieve a random access of the regions of interest^6,7^. Moreover, the axial scanning performance of MPEF microscopes can be improved by utilizing electrically tunable lenses^8,9^ or ultrasound lenses. Tunable acoustic gradient index of refraction (TAG) lenses can perform scanning at frequencies in the range of 100–500 kHz^10–14^. Thus, the imaging speed can reach frame rates as high as 1 kHz and volumetric imaging rates of around 56 Hz, which depends on the camera frame rate^15^. Spatial separation and optical time delay designs can achieve imaging speeds of up to 3000 fps by exploiting free-space angular-chirp-enhanced delay technology^16^. Similarly, scan multiplier units interfaced with two-photon microscopes can provide line-scan rates of around 592 kHz and imaging speeds as high as 16,000 fps^17^. However, since the volumetric images are constructed sequentially pixel-by-pixel in 3D, the total volumetric imaging rate is still restricted by the speed of the scanner. Simultaneous scanning provides a further opportunity for increasing the imaging speed. For example, the use of two RMs in the scanning process can potentially reach frame rates at kilohertz level^18^. The present group previously proposed a MPEF microscope with a volumetric imaging rate of 30 vps incorporating a TAG lens and a RM^19^. More recently, Deguchi et al.^20^ presented a confocal microscopy system based on Lissajous scanning, which achieved an imaging speed of 5000 fps. However, even though MPEF microscopy provides deep penetration, the limited pulse number restricts the image voxel number and MPEF signal level under ultrahigh speed imaging. Nonetheless, this limitation can be mitigated by increasing the repetition rate through the use of repetition rate multipliers^21^.

The present study therefore proposes a MPEF microscope for rapid volumetric bioimaging, in which a TAG lens is used to perform scanning in the *z*-axis direction, while a RM and GM scanner are used to perform scanning in the *x*- and *y*-axis directions, respectively. The proposed dual-resonant scanning multiphoton (DRSM) microscope provides high temporal resolution with low signal crosstalk, high sensitivity, and a high volumetric imaging rate. Currently, a frame rate of around 8000 Hz can be obtained in the *x–z* plane for an image region with a size of 256 × 80 pixels, while a volumetric imaging rate of more than 30 Hz can be achieved for a large image volume of 343 × 343 × 120 μm^3^ with 256 × 256 × 80 voxels. The practical feasibility of the proposed microscope is demonstrated by observing the mushroom body (MB) structure of a drosophila brain (OK-107) and performing 3D dynamic observations of moving 10-μm fluorescent beads.

## System design and setup

### Overall system setup

**Figure 1:**
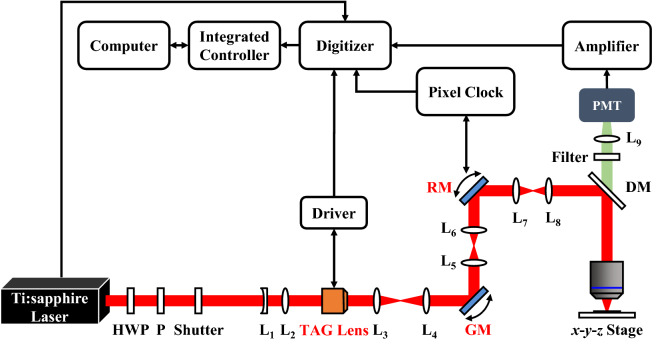
Overall setup of rapid volumetric DRSM microscope.

Figure 1 illustrates the schematic configuration of the rapid volumetric DRSM microscope. As shown, the illumination source is provided by a femtosecond Ti:sapphire laser (Tsunami, Spectra-Physics, USA) with a repetition rate of 80 MHz. The laser light is passed through a half-wave plate (HWP) and polarizer (P) to adjust the laser power and achieve a horizontal polarization state. A mechanical shutter (LS3, Uniblitz, USA) is placed to block the laser beam when imaging is not in progress in order to avoid photodamage and photobleaching of the sample. The laser beam is then aligned and expanded using two lenses (L_1_ and L_2_) before entering the scanning system, which comprises a TAG lens, a GM scanner (6215H, Cambridge, USA), a RM scanner (CRS series, Cambridge, USA), and three relay lens systems (L_3_, L_4_, L_5_, L_6_, L_7_, and L_8_). The TAG lens is used to scan the sample in the *z*-axis direction with a driving resonant frequency at around 456 kHz. In the meanwhile, the RM scanner with a driving resonant frequency at 8 kHz and the GM scanner are utilized to scan the sample in the *x*- and *y*-axis directions, respectively. The laser beam reflected from the RM is further reflected by a dichroic mirror (DM) (FF670-Di01, Semrock, USA) to cover the back aperture of an objective lens (W Plan-Apochromat 20X/1.0, Carl Zeiss, Germany). After passing through the objective lens, the beam is focused on the sample to excite MPEF. The resulting fluorescence signal passes through a band-pass filter and a focusing lens (L_9_) before captured by a photomultiplier tube (PMT) (H7422-40, Hamamatsu, Japan), where it is converted into an electrical signal for further processing.

The DRSM imaging system is integrated in an upright optical microscope (Axio Imager 2, Carl Zeiss, Germany) with a programmable motorized three-axis stage (HEP4AXIM/B ProScan, Prior Scientific, UK) and an objective scanner (PD72Z4CAA, Physik Instrumente, UK) with a 400 μm travel range. The overall instrument communication and control is implemented using a self-written LabVIEW program built on an integrated controller PXI chassis (PXI-1033, National Instrument, USA) consisting of a FlexRIO FPGA module (PXI-7952R, National Instrument, USA), a PXI multifunction reconfigurable I/O module with eight analog-to-digital converters (ADCs) (PXI-7842R, National Instrument, USA), and a digitizer (NI 5761, National Instrument, USA). The pulse train signal (i.e., the repetition rate) of the laser is used as the external clock of the digitizer such that each cycle loop in the FPGA is locked with the repetition rate of the laser. Furthermore, the pulse train signal is used as a synchronization clock to trigger the ADCs in such a way as to sample the fluorescent signal at the rate of one laser pulse per pixel. Since the pulse train signal and fluorescent signal are effectively paired with a constant time delay, the efficiency of the sampling process is greatly improved. The sampling process is determined by the synchronization TTL signal of the TAG lens and the pixel-enable signal of the RM scanner produced by the pixel clock board. Since the RM motion trajectory is nonlinear, the pixel-enable of the RM is utilized, and thus only the near linear region of the RM motion trajectory is used for sampling. The scanning data are transmitted to a computer via direct memory access (DMA) first in first out (FIFO) channels. In addition, the PXI-7842R controls the GM and logical operational function.

### Synchronization design

In general, synchronization plays a vital role in achieving ultrahigh speed imaging in rapid volumetric DRSM microscopes. In the present system, to achieve a volumetric imaging rate of up to 30 vps, the frame rate of each slice image should exceed 8000 fps, i.e., a maximum exposure time of 0.125 ms. That is, each slice image is constructed using just 10,000 laser pulses when a femtosecond laser with a repetition rate of 80 MHz is used to excite the MPEF signal.

**Figure 2:**
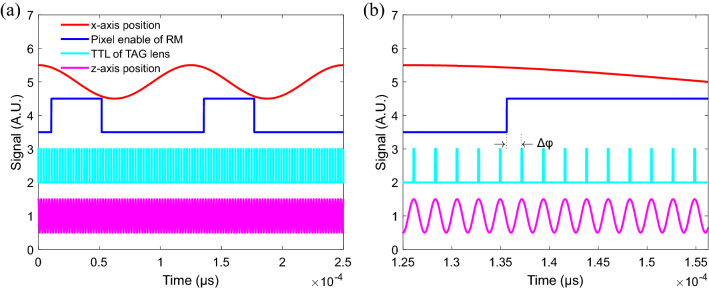
(**a**) Schematic diagram showing signals for *x*-axis scanning position, pixel enable of RM, TTL of TAG lens, and *z*-axis scanning position. Note that the red and blue lines show the *x*-axis scanning position and pixel enable signal from the RM driver. The cyan line shows the TTL signal of the TAG lens produced by the DrvKit 3.3 driver. The magenta line shows the *z*-axis scanning position relative to the scanning position of the TAG lens. (**b**) Close-up view of relationship among four signals. Note the presence of a phase delay, Δφ, between the pixel enable signal and the TTL signal.

Figure 2a shows the relationship among the signals produced by the TAG lens and RM, respectively. The red and blue lines show the relative *x*-axis scanning position and pixel enable signal of the RM, respectively. (Note that the pixel enable signal is corrected within the linear portion of the RM scanning region.) The cyan line shows the TTL signal of the TAG lens produced by its electric driver (DrvKit 3.3 driver in Fig. 5), where this signal has a frequency equal to the resonant frequency of the lens (~ 456 kHz in the present design). Finally, the magenta line shows the relative *z*-axis scanning position of the TAG lens, which measured by a self-built confocal detector to confirm the stability of the lens. As shown in Fig. 2b, the *z*-axis relative scanning position of the TAG lens varies stably with a simple harmonic motion. However, the scanning *x*–*z* trajectory of the proposed MPEF microscope has the form of a Lissajous curve since the TAG lens and RM perform synchronized harmonic scanning. Moreover, the resonant frequencies of the two devices are not perfectly divisible by one another. Therefore, the obtained MPEF image has a non-uniform pixel distribution under ultrahigh speed imaging. Nonetheless, by estimating the phase delay, Δφ, of the TTL signal of the TAG lens relative to the pixel-enable signal of the RM (see Fig. 2b), the Lissajous scanning pattern can still be accurately predicted. Therefore, the *z*-axis relative scanning position corresponding to the sampled MPEF signal can be obtained, and thus the captured images can be reconstructed.

Accordingly, in the present DRSM microscope, the repetition rate (i.e., pulse train signal) of the femtosecond laser is used as a trigger to both acquire the MPEF signal and simultaneously record the starting position of the scanning process in the *z*-axis direction. When the pixel enable signal is “ON”, the digitizer starts to acquire the MPEF signal with a sampling rate of 80 MHz (i.e., the repetition rate of the laser). The starting position of the scanning process in the *z*-axis direction is not the same in each frame since the resonant frequencies of the TAG lens and RM are not perfectly divisible. However, as described above, the starting position in the *z*-axis direction can be obtained by calculating the phase delay, Δ*φ*. Due to the fixed sampling rate used in the DRSM system, the phase difference between two pixels is fixed. The scanning position (i.e., pixel) corresponding to the MPEF signal can thus be easily calculated from its phase difference. In particular, due to the simple harmonic motion of the TAG lens and RM scanning trajectories, the sampled positions in the *z*- and *x*-axis directions, respectively, are both related to the phase delay of the corresponding pixel, which is equal to the current phase difference between the pixel enable signal and the TTL signal of the TAG lens. Notably, the sampled position in the *x*-axis direction is almost the same for each frame since the pixel enable signal is constant. In other words, the starting position is determined mainly by the pixel enable signal. However, the phase delay of the *z*-axis direction is variable (i.e., the Lissajous curve pattern is variable) for each frame, and hence the sampled position in the *z*-axis direction for each frame must be calculated from the phase delay.

For the optical setup considered in the present study, the imaging region has a size of around 343 μm (256 pixels) in the *x*-axis direction and 120 μm (80 pixels) in the *z*-axis direction. In reconstructing the *x–z* sectional images, the acquired MPEF signals are mapped to the corresponding nearest positions in the imaging array with 256 × 80 pixels. Since the RM scanner has a driving resonant frequency of 8 kHz, the frame rate of the *x–z* sectional image can reach approximately 8000 fps. Assuming that the image region has a size of 343 μm within 256 pixels in both the *x*-axis direction and the *y*-axis direction, the volumetric imaging rate of the proposed DRSM microscope can reach approximately 30 vps with a volume size of 343 × 343 × 120 μm^3^ which is corresponding to an image size of 256 × 256 × 80 voxels.

## Experimental results and discussions

### Calibration of DRSM microscope

**Figure 3:**
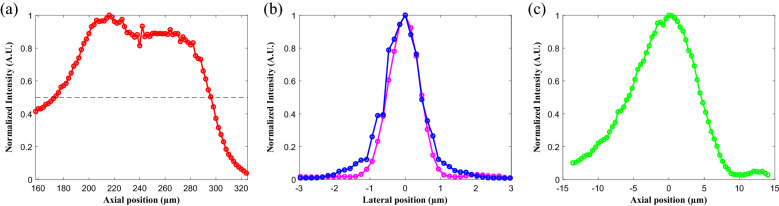
(**a**) Axial intensity profile of fluorescent thin film while undergoing axial displacement by stage. Axial scanning range is approximately 120 μm at full width at half maximum (FWHM). (**b**) Spatial resolutions are 1.02 μm and 1.18 μm in *x*-axis (magenta) and *y*-axis (blue), respectively. (**c**) Axial resolution is 10.72 μm.

The rapid volumetric DRSM microscope was calibrated using a fluorescent thin film and fluorescent beads as samples. In both cases, the samples were driven by a programmable motorized three-axis stage (HEP4AXIM/B ProScan, Prior Scientific, UK). The maximum field of view of the proposed system design was around 343 × 343 μm^2^ based on the chosen objective lens (W Plan-Apochromat 20X/1.0, Carl Zeiss, Germany) and TAG lens (TAG Lens 2.0, TAG Optics Inc., USA) with a resonant frequency of 456 kHz. Figure 3a shows the captured intensity profile of the fluorescent thin film when driven axially by the stage. It is seen that the axial scanning range is around 120 μm when driving the TAG lens at 456 kHz. Consequently, the ability of the proposed DRSM microscope to achieve a large image region of 343 × 343 × 120 μm^3^ is confirmed. The spatial resolution of the proposed microscope was evaluated using a single 200-nm fluorescent bead (F-8888, Thermo Fisher Scientific, USA) fixed in agarose gel. In the optical design of the microscope, the TAG lens was conjugated with the back aperture of the objective. Moreover, the effective aperture of the TAG lens was around 1.8 mm with 20 diopters at the resonant frequency of 456 kHz. To achieve the axial scanning range of 120 μm described above, the laser beam emerging from the TAG lens was only expanded by around 3.33 times by the relay lens systems (see Fig. 1). The resulting coverage of the back aperture of the 20X objective, which is 12.4 mm in diameter, was found to be just 48% (i.e., 1.8 × 3.33/12.4). Therefore, the lateral and axial resolutions of the DRSM microscope were theoretically degraded by up to 1/0.48 and 1/0.48^2^ times, respectively. To calibrate the spatial resolution of the microscope, the spatial sampling periods in the *x*–*y* plane and *z*-axis direction were set as 0.16 μm and 0.4 μm, respectively. The microscope was then used to observe a single 200-nm fluorescent bead fixed in agarose gel. As shown in Fig. 3b, the resulting spatial resolutions in the *x*-axis and *y*-axis directions (i.e., the lateral resolution) were 1.02 μm and 1.18 μm, respectively, at the full width at half maximum (FWHM). In addition, the spatial resolution in the *z*-axis direction (i.e., the axial resolution) was 10.72 μm, as shown in Fig. 3c.

When the objective was increased to 40X, the laser beam filled nearly the entire back aperture (i.e., around 5.94 mm) of the objective (W Plan-Apochromat 40X/1.0, Carl Zeiss, Germany). The axial resolution was thus improved from 10.72 to 2.78 μm, which was close to the theoretical value for the 40X objective. However, the axial scanning range was significantly reduced from 120 μm (Fig. 3a) to just 31.3 μm. Thus, an alternative approach was attempted in which the TAG lens was simply operated at a lower resonant frequency of 143 kHz with 1.5 diopters. The effective aperture of the TAG lens then became 5.5 mm, and the laser beam, with a size of 18.3 mm (i.e., 5.5 × 3.33), completely covered the back aperture of the 20X objective. However, the axial scanning range was reduced yet further to 9 μm. Hence, a trade-off was found to exist between the axial resolution and the axial scanning range. Notably, the frame rate was unchanged despite the use of a lower TAG resonant frequency. However, the *x*-axis image region contained only 17 TAG resonant scanning lines. Thus, compared with the original operating mode of 456 kHz with 57 TAG resonant scanning lines, the *x*-axis scanning resolution was degraded by more than 3 times.

The aim of the DRSM microscope proposed in this study was to realize a long axial scanning range under a concise dual-resonant scanning setup with only a single pass through the TAG lens^14^. Hence, the results presented above indicate that a trade-off must be made among the axial scanning range, the axial spatial resolution, and the *x*-axis scanning resolution^22^.

### High-speed imaging performance and restriction

**Figure 4:**
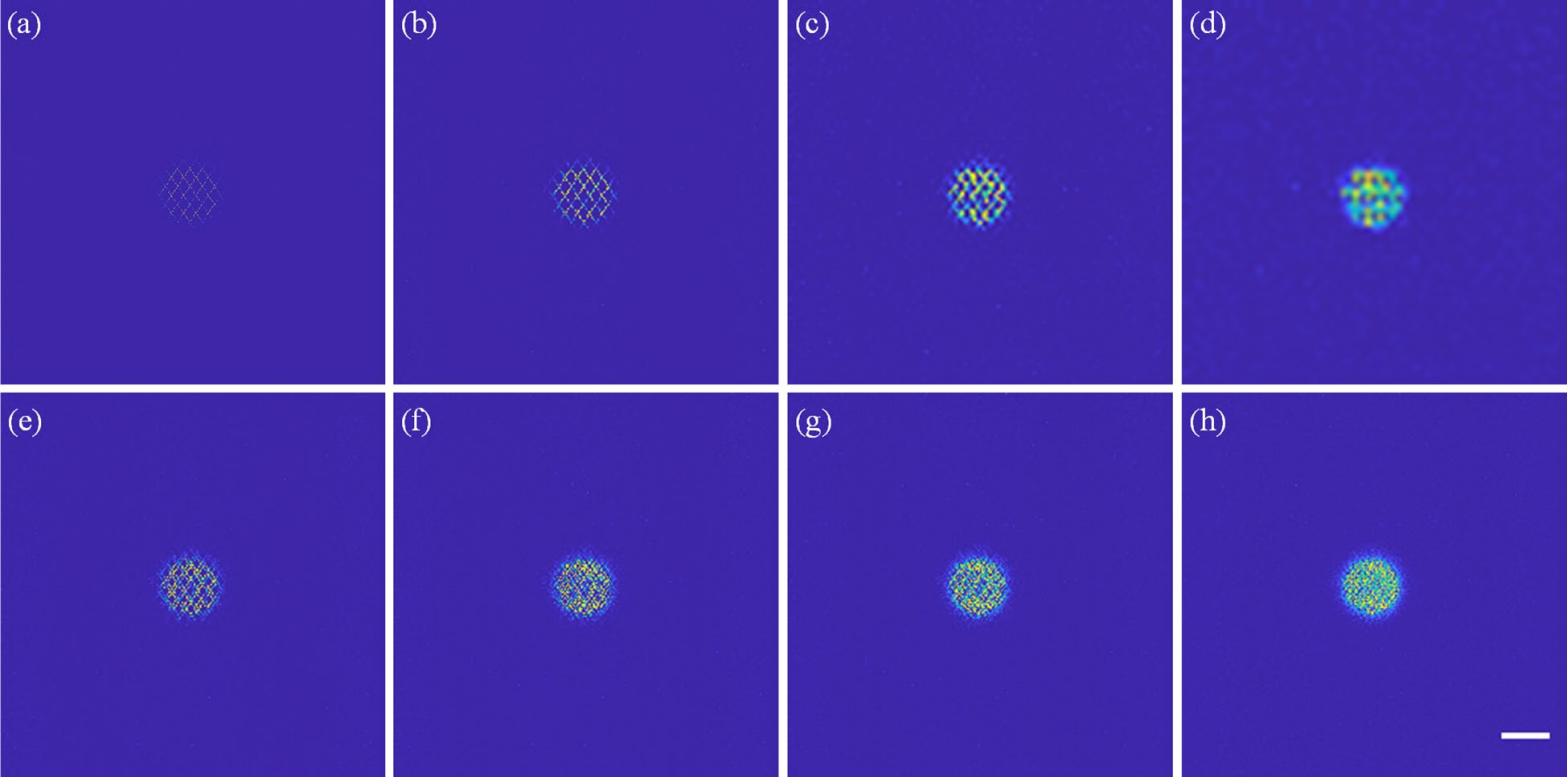
(**a**–**d**) *x*–*y* cross-sectional images of 10-μm fluorescent bead captured at 30 vps with image sizes of 512 × 512, 256 × 256, 128 × 128, and 64 × 64 pixels, respectively, corresponding to pixel ratios of 0.08, 0.24, 0.68 and 0.94. (**e**–**h**) 2, 3, 5, and 10 accumulated images from *x*–*y* cross-sectional images with 256 × 256 pixels and pixel ratios of 0.42, 0.55, 0.7, and 0.84, respectively. Scale bar: 10 μm.

Figure 4a–h show *x*–*y* cross-sectional images captured of a 10-μm fluorescent bead (F-8836, Thermo Fisher Scientific, USA) with an *x*–*y* plane image region of 80 × 80 μm^2^. Figure 4a–d present the images captured with *x*–*y* image sizes of 512 × 512, 256 × 256, 128 × 128 and 64 × 64 pixels, respectively, which is corresponding to pixel ratios of 0.08, 0.24, 0.68 and 0.94, for a constant volumetric imaging rate of 30 vps. Figure 4a and b contain a large number of missing pixels, and hence it is difficult to pinpoint the center of the bead precisely. Furthermore, even though Fig. 4c and d have larger pixel ratios, the lateral scanning resolutions are reduced by 4 and 8 times compared to that of Fig. 4a, respectively. Accordingly, an attempt was made to improve the imaging performance by accumulating multiple volumetric images. Figure 4e–h show the results obtained when accumulating 2, 3, 5, and 10 *x*–*y* cross-sectional images with a size of 256 × 256 pixels in the *x*–*y* plane (i.e., effective volumetric imaging rates of 15, 10, 6, and 3 vps, respectively) and pixel ratios of 0.42, 0.55, 0.7, and 0.84. The image quality of Fig. 4e–h is greatly improved compared to that of Fig. 4a–d. However, the image accumulation approach is infeasible for 3D dynamic observations due to the corresponding reduction of the volumetric imaging rate.

**Figure 5:**
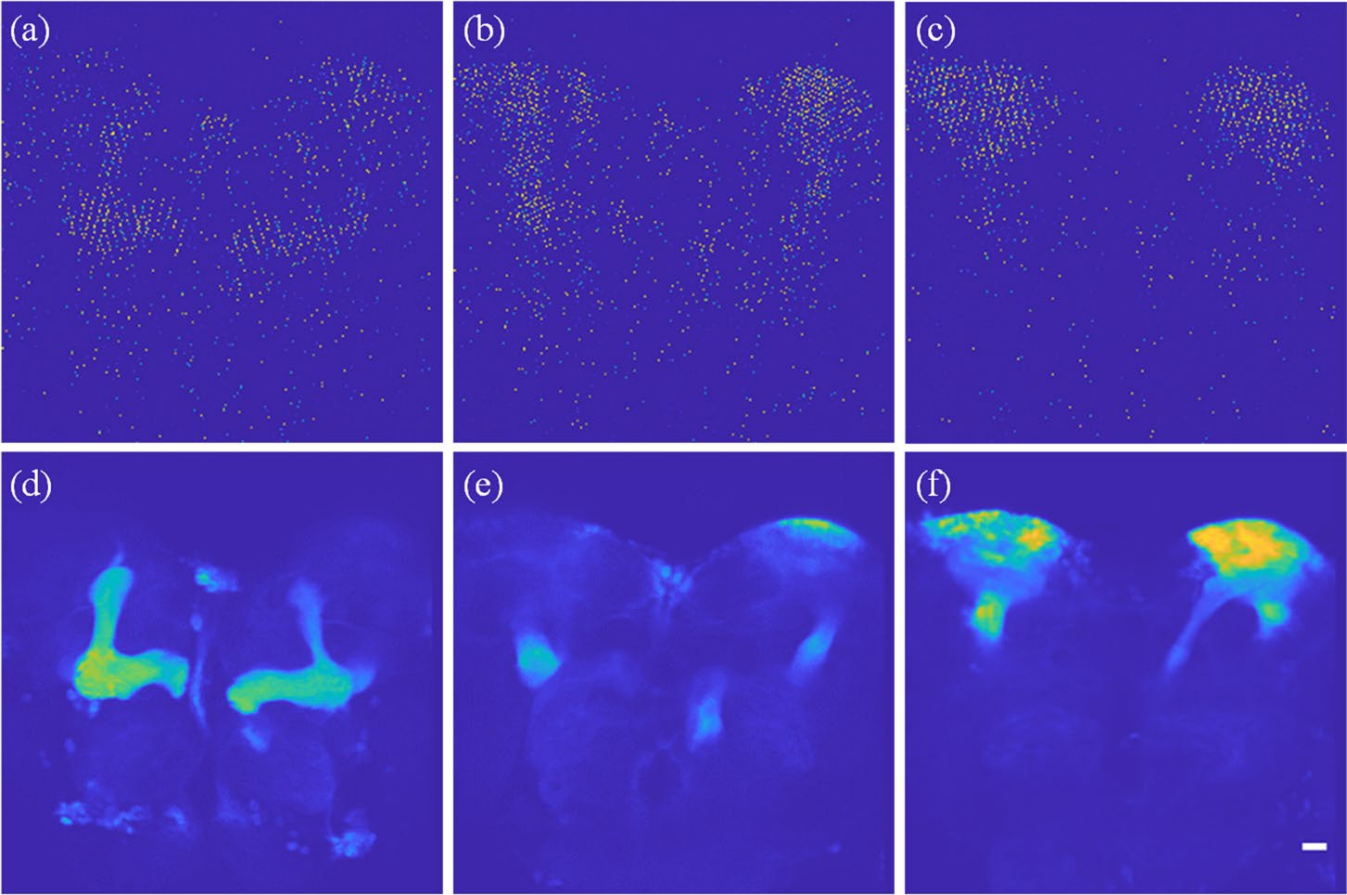
(**a**–**c**) *x–y* cross-sectional images of MB structure obtained at − 30-μm, 0-μm, and 30-μm depth layers, respectively. (**d**–**f**) Composite images obtained by accumulating 100 volumetric images at corresponding depth layers. Note that the image region is 343 × 343 × 120 μm^3^, the volumetric imaging rate is 30 vps, and the image size is 256 × 256 × 80 voxels. Scale bar: 20 μm. Video 1 shows the corresponding volumetric images of the MB structure without (i.e., original) and with 100-volumetric-image accumulation, respectively.

The bioimaging performance of the DRSM microscope was further evaluated using the mushroom body (MB) structure of a drosophila brain. The transgenic line of drosophila melanogaster demonstrated in this study is OK-107, which carries eGFP in the whole MB. The chosen samples were placed in 4 °C for immobilization before dissecting. Under the dissection microscopy, the drosophila brain was pulled out carefully with fine tip forceps. After the dissection, the brains were collected and fixed via 1.2% PFA. The fixed brains were mounted on a microscope slide with a spacer and filled with mounting medium. The sample was stored at 4 °C and prevented from light before performing under the DRSM imaging. The brain sample had a thickness of around 100 μm, and thus its entire volume was observed by the DRSM microscope with an image region size of 343 × 343 × 120 μm^3^ at volumetric rate of 30 vps. Figure 5a–c present the *x–y* cross-sectional images of the brain obtained with 256 × 256 × 80 voxels at depth layers of − 30 μm, 0 μm, and 30 μm, respectively. Figure 5d–f show the corresponding results obtained when accumulating 100 volumetric images at each plane, respectively. Based on the *x*–*y* image size of 256 × 256 pixels in Fig. 5a–c, the pixel ratio is 0.24 in every case. However, the intensity distributions of the two series of volumetric images show a broadly similar morphology. Video 1 shows the corresponding volumetric images of the MB structure without (i.e., original) and with 100-volumetric-image accumulation, respectively.

To maintain a rapid volumetric rate with a large image volume and large image size given only a limited number of laser pulses for each image, the voxel number and SNR must be significantly increased. This can be achieved in a direct fashion by simply increasing the repetition rate of the laser^21^. Alternatively, the resonant frequency of the TAG lens can be reduced^14,22^. Furthermore, in a preliminary stage of this study, we found the SNR to be ~ 7 dB for one pulsed laser fluorescent signal per voxel with R6G solution as the sample. Due to the limited signal level and low photon budget, the potential use of this rapid volumetric multiphoton imaging technique for in vivo studies needs to be further investigated. Also, the problem of Lissajous patterning residuals caused by dual-resonant scanning must be overcome. Previous studies have suggested that both issues can be addressed by performing image inpainting and denoising using conventional or deep learning approaches^22,23^.

### Dynamic imaging performance

The DRSM microscope was found to require 0.032 s to obtain each volumetric image, that is, the volumetric imaging rate was higher than 30 vps. The dynamic 3D imaging performance of the microscope was evaluated by observing the simple harmonic motion of the 10-μm fluorescent bead in real time as it underwent periodic displacement along the *z*-axis at various frequencies. In performing the imaging trials, the bead immobilized in agarose gel was placed on a piezoelectric stage and driven at five different temporal frequencies of 1, 5, 10, 15, and 20 Hz, respectively. For each driving frequency, the volumetric images of the fluorescent bead were captured continuously for a period of around 3 s with a volumetric imaging rate of 30.6 vps with an image region size of 80 × 80 × 120 μm^3^ and 256 × 256 × 80 voxels. Compared with the 343 × 343 μm^2^ image region in the *x*–*y* plane, the 80 × 80 μm^2^ smaller image region with the same 256 × 256-pixel size was then used to lessen around 4 × 4-time spatial sampling period (i.e., 80/256 vs. 343/256) of the volumetric images to enable the peak intensity positions corresponding to the centers of the fluorescent bead at different time instances to be more reliably determined.

**Figure 6:**
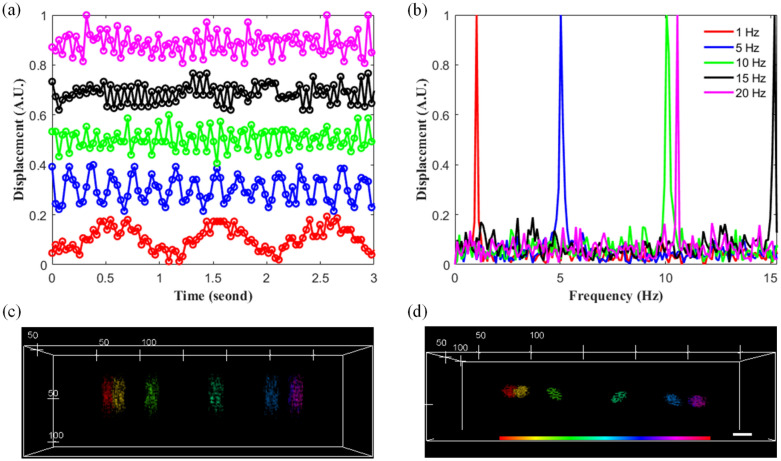
(**a**) Motion displacements and (**b**) motion spectra of 10-μm fluorescent beads driven at vibrational frequencies of 1 Hz, 5 Hz, 10 Hz, 15 Hz, and 20 Hz, respectively. (**c**,**d**) Volumetric images of 10-μm fluorescent bead recorded at six different time instances in *x–z* and *x–y* views, respectively. Scale bar: 20 μm. Video 2 shows volumetric videos of the 10-μm fluorescent bead for an image region size of 343 × 80 × 120 μm^3^ and volumetric sizes of 256 × 256 × 80 voxels for 30 vps and 256 × 64 × 80 voxels for 120 vps, respectively.

Figure 6a shows the displacements of the fluorescent bead under the five driving frequencies. For each frequency, the vibrational frequency of the bead was estimated from the displacement signal via fast Fourier transform. The normalized temporal spectra of the bead displacements are shown in Fig. 6b. It is seen that the 20 Hz vibration displacement is aliased to 10.6 Hz (i.e., 30.6–20 Hz). However, for frequencies lower than 15.3 Hz (i.e., one half of the volumetric imaging rate (30.6 vps)), the DRSM microscope accurately tracks the bead displacement as the piezoelectric stage oscillates. In other words, the dynamic imaging performance of the DRSM microscope is confirmed.

The practical feasibility of the DRSM microscope was further evaluated by observing the high-speed movement of the 10-μm fluorescent bead for an image region of 343 × 80 × 120 μm^3^ within 256 × 256 × 80 and 256 × 64 × 80 voxels for volumetric imaging rates of 30 and 120 vps, respectively. In both cases, the bead was moved over a distance of 200 μm in the left to right *x*-axis direction at a speed of 6000 μm/s. Figure 6c and d present the 30-vps volumetric images of the fluorescent bead at 6 different time instances (0, 0.032, 0.064, 0.096, 0.128, and 0.160 s) in the *x–z* and *x–y* views, respectively. In the *x–z* view, the morphology of the bead is enlarged as a result of the bead motion in the *x*-axis direction and the axial resolution of 10.72 μm. By contrast, in the *x–y* view, the morphology of the fluorescent bead is obliquely elongated due to the *x*-axis moving direction and *y*-axis GM scanning direction. The results presented in Fig. 6c and d show that the stage has a slower speed initially, a faster speed in the middle of the displacement range (as shown by a larger bead elongation), and a slower speed toward the end of the displacement range (200 μm). To eliminate the morphology enlargement and elongation effects in the *x*-axis direction, the volumetric imaging rate was boosted to 120 vps. The higher imaging rate eliminated the morphology enlargement and elongation, but resulted in only a sparsely-sampled volume. However, this drawback can be overcome by using either conventional or deep learning inpainting and denoising approaches^23,24^. Video 2 shows volumetric video sequences of the 10-μm fluorescent bead at imaging rates of 30 vps and 120 vps, respectively. The results confirm that a high volumetric imaging rate is necessary for fast moving particles.

Compared with TF widefield multiphoton and one-photon excitation, multiphoton point scanning technology provides the potential to achieve a greater penetration depth. Thus, the development of high-speed multiphoton microscopy systems continues to attract attention in the literature^25–27^. The present study explores the feasibility for performing high-speed volumetric multiphoton imaging using a dual-resonant scanning approach. Moreover, the details of the dual-resonant technique, and its advantages and disadvantages for fast volumetric multiphoton imaging, are presented and discussed.

## Conclusions

This study has presented a DRSM microscope incorporating a TAG lens and a RM for rapid volumetric imaging. It has been shown that the microscope achieves a frame rate of approximately 8000 fps in the *x*–*z* plane given an image region of 343 × 120 μm^2^, corresponding to an image size of 256 × 80 pixels. The volumetric image rate is thus around 30 vps for an image volume of 343 × 343 × 120 μm^3^, corresponding to an image size of 256 × 256 × 80 voxels. The results obtained using a 10-μm fluorescent bead as the sample have shown that the DRSM microscope can observe dynamic motions with vibrational frequencies of up to 15 Hz without image aliasing effects. Moreover, the practical feasibility of the microscope has been demonstrated by bioimaging the MB structure of a drosophila brain. To maintain a rapid volumetric rate with a large image volume and large voxel number, it is necessary to mitigate the severe negative signal-to-noise ratio caused by the large number of missing voxels through the use of conventional or deep learning image inpainting and denoising methods. Nonetheless, the present results suggest that the rapid volumetric DRSM microscope proposed in this study provides a powerful tool for exploring multiscale signaling dynamics.

## Supplementary Information

Supplementary Legends.

Supplementary Video 1.

Supplementary Video 2.

## Data Availability

The datasets used and analyzed in the current study are available from the corresponding author on reasonable request.
