# Supplementary material for: Dual-resonant scanning multiphoton microscope with ultrasound lens and resonant mirror for rapid volumetric imaging
Source: Sci Rep. 2023 Jan 4;13:161. doi: 10.1038/s41598-022-27370-w (PMC9813223; doi:10.1038/s41598-022-27370-w)
Supplement: Supplementary file 1 — Supplementary Legends. [file 41598_2022_27370_MOESM1_ESM.docx]

**Video Legends**

**Video 1.** The volumetric images of the MB structure without (i.e., original) and with 100-volumetric-image accumulation corresponding to Figs. 3(a)-3(c) and Figs. 3(d)-3(f), respectively.

**Video 2.** The volumetric videos of the 10-μm fluorescent bead for an image region size of 343×80×120 μm^3^ and volumetric sizes of 256×256×80 voxels for 30 vps and 256×64×80 voxels for 120 vps, respectively.
